# Silica formation with nanofiber morphology via helical display of the silaffin R5 peptide on a filamentous bacteriophage

**DOI:** 10.1038/s41598-017-16278-5

**Published:** 2017-11-24

**Authors:** In-Wong Song, Hyojung Park, Jung Han Park, Hyunook Kim, Seong Hun Kim, Sung Yi, Justyn Jaworski, Byoung-In Sang

**Affiliations:** 1 Department of Fuel Cell and Hydrogen Technology, Hanyang University, 222 Wangshimni-ro, Seongdong-gu, Seoul, 04763 Republic of Korea; 2 Department of Chemical Engineering, Hanyang University, 222 Wangshimni-ro, Seongdong-gu, Seoul, 04763 Republic of Korea; 3 Science&Technology Policy Coordination Division, Ministry of Science, ICT and Future Planning, 47 Gwanmun-ro, Gwacheon-si, Gyeonggi-do, 13809 Republic of Korea; 4 Department of Environmental Engineering, 163 Seoulsiripdaero, Dongdaemun-gu, The University of Seoul, Seoul, 02504 Republic of Korea; 5 Department of Organic and Nano Engineering, Hanyang University, 222 Wangshimni-ro, Seongdong-gu, Seoul, 04763 Republic of Korea; 6 Department of Bioengineering, University of Texas at Arlington, 500 UTA Blvd., Arlington, TX 76019 USA

## Abstract

Biological systems often generate unique and useful structures, which can have industrial relevance either as direct components or as an inspiration for biomimetic materials. For fabrication of nanoscale silica structures, we explored the use of the silaffin R5 peptide from *Cylindrotheca fusiformis* expressed on the surface of the fd bacteriophage. By utilizing the biomineralizing peptide component displayed on the bacteriophage surface, we found that low concentrations (0.09 mg/mL of the R5 bacteriophage, below the concentration range used in other studies) could be used to create silica nanofibers. An additional benefit of this approach is the ability of our R5-displaying phage to form silica materials without the need for supplementary components, such as aminopropyl triethoxysilane, that are typically used in such processes. Because this method for silica formation can occur under mild conditions when implementing our R5 displaying phage system, we may provide a relatively simple, economical, and environmentally friendly process for creating silica nanomaterials.

## Introduction

The versatility of silica can be seen in the fabrication of diverse industrial components, including biomedical, catalytic, cosmetic, and pharmaceutical applications, in addition to its use in separation media, detergents, and coatings^1,2^. Many approaches, including chemical, biological, and hybrid processes, enable the production of a variety of silica structures. Among the chemical processes, Stober’s method has been widely used in multiple industries;^3^ however, biological and hybrid processes are becoming more meaningful for sustainable development. In the existing literature, filamentous bacteriophages have been utilized in the fabrication of silica structures. In such approaches, phages are coated with inorganic compounds, such as tetramethyl orthosilicate (TMOS) and tetraethyl orthosilicate (TEOS) among other silica precursors. These hybrid phage/silica structures have been applied in the areas of catalysis and electronics, wherein they serve as functional nanoparticles with the capability of self-assembly^4–7^.

In our hybrid strategy of silica structure fabrication, we utilized the silaffin R5 peptide sequence from *Cylindrotheca fusiformis* ^8–11^. In previous reports, the silaffin R5 peptide has been shown to generate spherical type silica by self-assembly, and the precise structure depends on the species-specific sequence of origin. Moreover, silaffin R5 peptide enables silicification above pH 7.0 and at room temperature^9,12^. The advantage of mitigating the need for high temperature processing demonstrates the eco-friendly benefits of using the R5 peptide sequence for silica formation under mild conditions. The properties and characteristics of the silaffin R5 peptide have recently been reported, including identification of the structure, as well as its mechanism of action highlighting the importance of the tetra amino acid motif (Arg-Arg-Ile-Leu) on the final silica morphology^13–15^. The use of various other silicifying peptide constructs, including repeating (Lys-Asp) units, chemically linked onto genetically modified tobacco mosaic virus have in some cases shown the ability to form 300 nm long silica rods after several days of mineralization^16^. In several related reports using the cationic polymer, poly-L-lysine, it has been proven that it may precipitate silica from precursor without the use of APTES (3-aminopropyl triethoxysilane); however in those cases the mineralized silica took on the geometries of spheres and spherical aggregates^14,17^. Looking to create an elongated silica nanofiber-like geometry as opposed nanospheres, we looked to the flexible fd bacteriophage particles as an alternative given their flexibility and length of nearly 1 um (and even longer for the case of multiple genome packaging into a single virus particle). In comparison with flagella or other biopolymeric fibers that have been used as templates in conjunction with APTES for nucleation and polymerization of TEOS for forming silica structures analogous to pearls on a string^18,19^, we instead explore here the use of fd bacteriophage with genetically modified R5 peptides displayed on the major coat for APTES-free formation of silica nanofiber structures. An additional benefit of the fd phage as a self-replicating template is that it offers facile production and purification.

**Figure 1:**
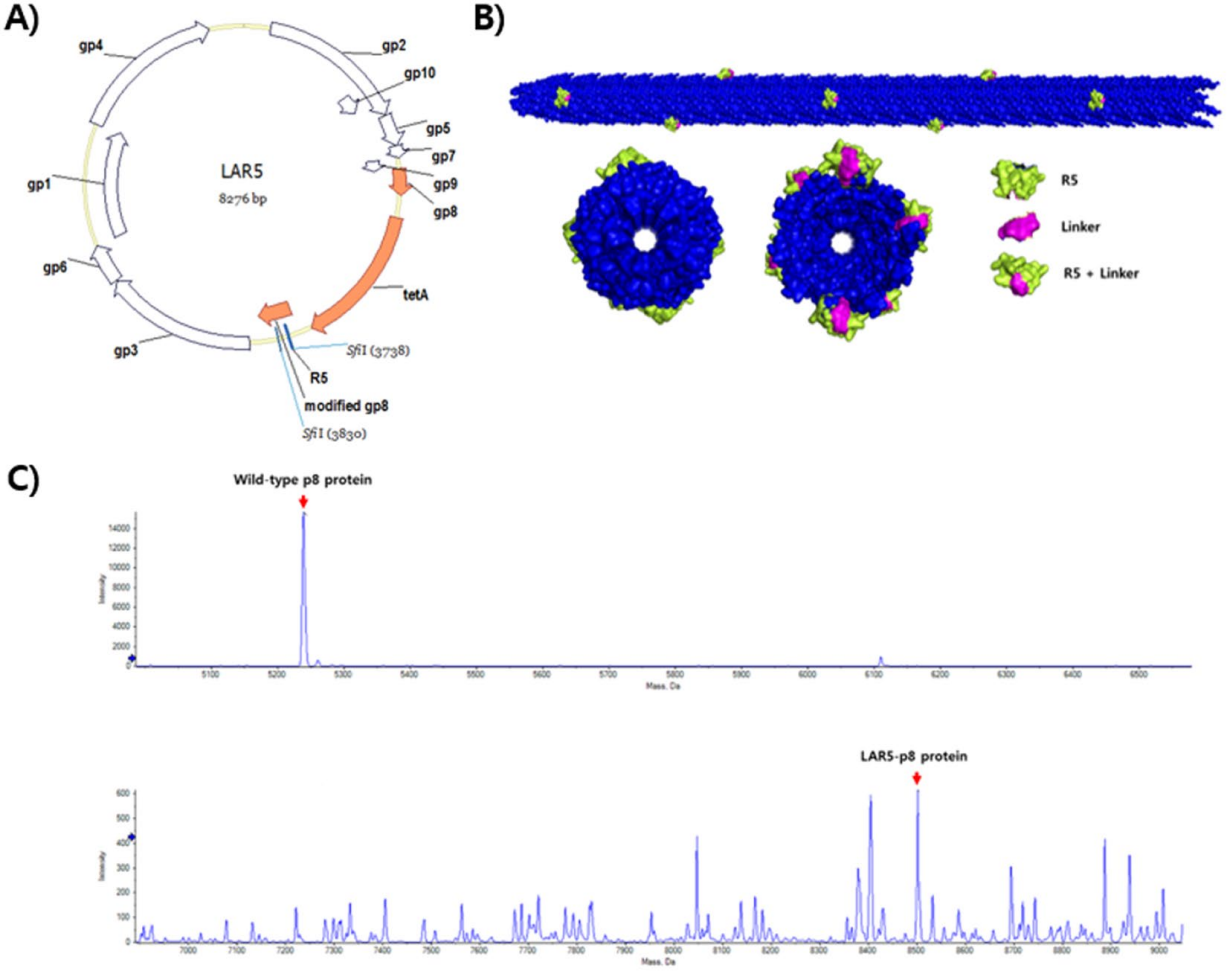
Schematic of phage used for silicification. (**A**) Plasmid map of LAR5 vector used for production of phage via the “type 88” phage system for simultaneous expression of the wild-type gp8 and the R5 silaffin-construct-appended p8 referred to as modified gp8. (**B**) Concept of silaffin R5 peptide expression on the p8 major coat protein of the fd bacteriophage. (**C**) Mass spectrum of expressed R5 silaffin on phage p8 coat.

Our specific goal in this work was to produce a two-part hybrid system utilizing the nanofiber morphology and helical display of peptides via the filamentous bacteriophage, along with the silica formation properties of the silaffin R5 peptide. Specifically, the silaffin R5 peptide was expressed on the p8 protein coat surface of a bacteriophage via a linker segment in order to enable the construction of nanoscale silica (Fig. 1). This report is distinct from prior reports in that there was no need to utilize any supporting compounds, such as 3-aminopropyl triethoxysilane (APTES), which are typically used during synthesis to allow interactions with TEOS or TMOS. In our method, such supporting compounds are not necessary, because the silaffin R5 peptide facilitates amenable interactions with the silica precursors and allows for rapid silicification. Here, we describe our approach, which may provide an economical and environmentally friendly technique for silica nanoparticle fabrication.

## Results

### Production of the LAR5 phage

We used the ‘type 88’ vector in this study to enable expression of the R5 peptide in a stable manner on the bacteriophage p8 coat. According to previous reports, the length and sequence of non-natural p8 peptides dramatically influences their level of expression^20,21^. The ‘type 88’ fth1 vector was utilized to construct our phage system, in which a 19-amino acid R5 sequence amended to the p8 was expressed under the control of a Tac promoter. The type 88 construct is known to allow longer peptides to be more easily expressed, and thus, the co-expression of wildtype p8 along with copies of p8 containing a linker-attached silaffin R5 peptide was easily achieved. This resulting vector is referred to as LAR5 and was used throughout our experiments for production of LAR5 phage (Fig. 1 A). To control the extent of R5 phage present and the amount of phage titer, we adjusted the amount of isopropyl-1-thio-β-D-galactopyranoside (IPTG) used to induce expression. Determination of phage concentrations was carried out through UV absorbance measurements at 269 nm and 320 nm and was calculated on the basis of existing methodology^22^. The final LAR5 phage was stored at 13 mg/ml for further testing and characterization. To verify the extent of silaffin R5 expression on the p8 coat, we utilized liquid chromatography mass spectrometry (LCMS). The results of mass spectrometry revealed the appearance of two different p8 proteins present on the phage sample, as was expected for the ‘type 88’ phage system. Specifically, the major peak was observed near 5240 Da and was indicative of the native p8 protein. The secondary peak was that of p8 bearing the linker-attached R5 and was located at approximately 8500 Da. From the LCMS results, we predicted that the amount of linker-attached silaffin R5 peptide on the phage’s p8 was 4.4% LAR5-p8 of the total copies of the major coat. Typically, the fd phage’s surface is composed of approximately 2700 copies of p8 protein, depending predominantly on the length of encapsulated DNA. Using the basis of 2700 p8 copies per phage particle, an estimated 120 copies of the LAR5-p8 will be expressed on each individual phage surface. The importance of utilizing the ‘type 88’ phage system is this ability to have effective packaging of otherwise unfavorable p8 modifications (i.e., due to charge or sterics) by co-inclusion of a large proportion of non-modified wild type p8 in order to accommodate formation of the viral particles.

### Silica formation by the LAR5 phage

**Figure 2:**
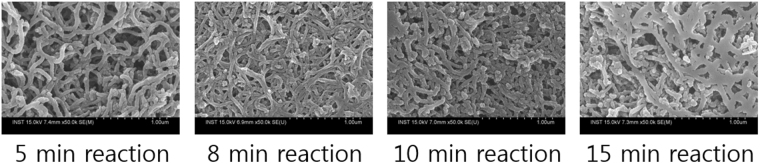
SEM images of silicified phage with increasing reaction times. LAR5 phage after increasing reaction times for silicification showing increasing thickness of the silica on the fiber-like phage particles.

According to previous reports, a silaffin protein linked to GFP expressed in *E. coli* has silicification activity^8,23^. The silaffin R5 peptide was confirmed to be a key component for silicification. To initially test the formation of silica particles around the phage as a function of reaction time, we examined a 1 mL reaction mixture comprised of 900 uL of 2 mg/mL of LAR5 phage with 2.5 mM APTES and 100 uL of 50 mM TMOS to find that silica particles could be formed in less than 15 minutes (Fig. 2). Utilizing the silica forming activity of the R5 peptide present on the surface of the LAR5 phage, we then examined 900 uL of 0.1 mg/ml of the LAR5 phage for silicification in the presence of 100 uL of 50 mM tetramethyl orthosilicate (TMOS) prehydrolyzed in 1 mM HCl. The LAR5 phage, when combined with the silica precursor, became slightly white within 2 minutes indicating silica condensation but was allowed to react for a total of 15 minutes. As a comparison, unmodified fth1 (which has no R5 moiety but instead the native p8 coat protein) was found to turn white after 90 minutes in the presence of APTES. These results suggest that the LAR5 phage surface displaying silaffin R5 peptides on the p8 coat protein initiate silicification much faster than the wildtype phage surface.

### SEM imaging of the surface of silicified LAR5 phages

**Figure 3:**
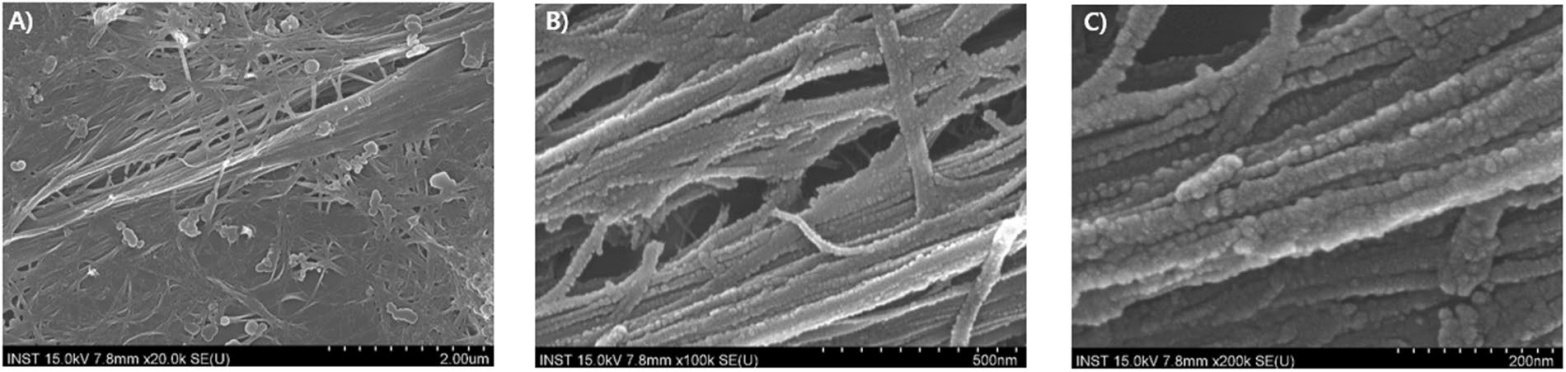
SEM images of silicified phage. LAR5 phage at (**A**) low (20k), (**B**) medium (100k), and (**C**) high (200k) magnification after silicification.

To analyze the morphology of the silica formation by LAR5 phage, we used scanning electron microscopy (SEM). From the SEM images, we confirmed the appearance of nanofiber silica hybrid structures with an approximate length of 1.0 μm (nearly the same length expected for LAR5 phage). The thickness was 30–50 nm, thus accounting for the silica formation around the phage, because filamentous phages are known to be only 6.6 nm in diameter. Separated structures and more ordered structure alignments were observable, some of which appeared as long lines greater than 1 μm. This increased length may be explained by the polyphage phenomenon in which multiple DNAs are packaged into a single phage particle, thereby resulting in a phage particle that is two or three times the typical length. When the samples were observed at high magnification, we observed that the fiber-like morphology was actually composed of individual nanoscale silica clusters. This result was desired, as the standalone R5 is known to nucleate spherical-structured silica. The formation of the spherical silica structures from the R5 peptide present on the p8 coat resulted in an increased diameter of the LAR5 phage particle composites relative to the 6.6 nm diameter of phage alone. Specifically, we could observe a 30–50 nm thick layer of silica deposited around the phage (Fig. 3).

### Comparison of LAR5 to fth1 phage with and without APTES

**Figure 4:**
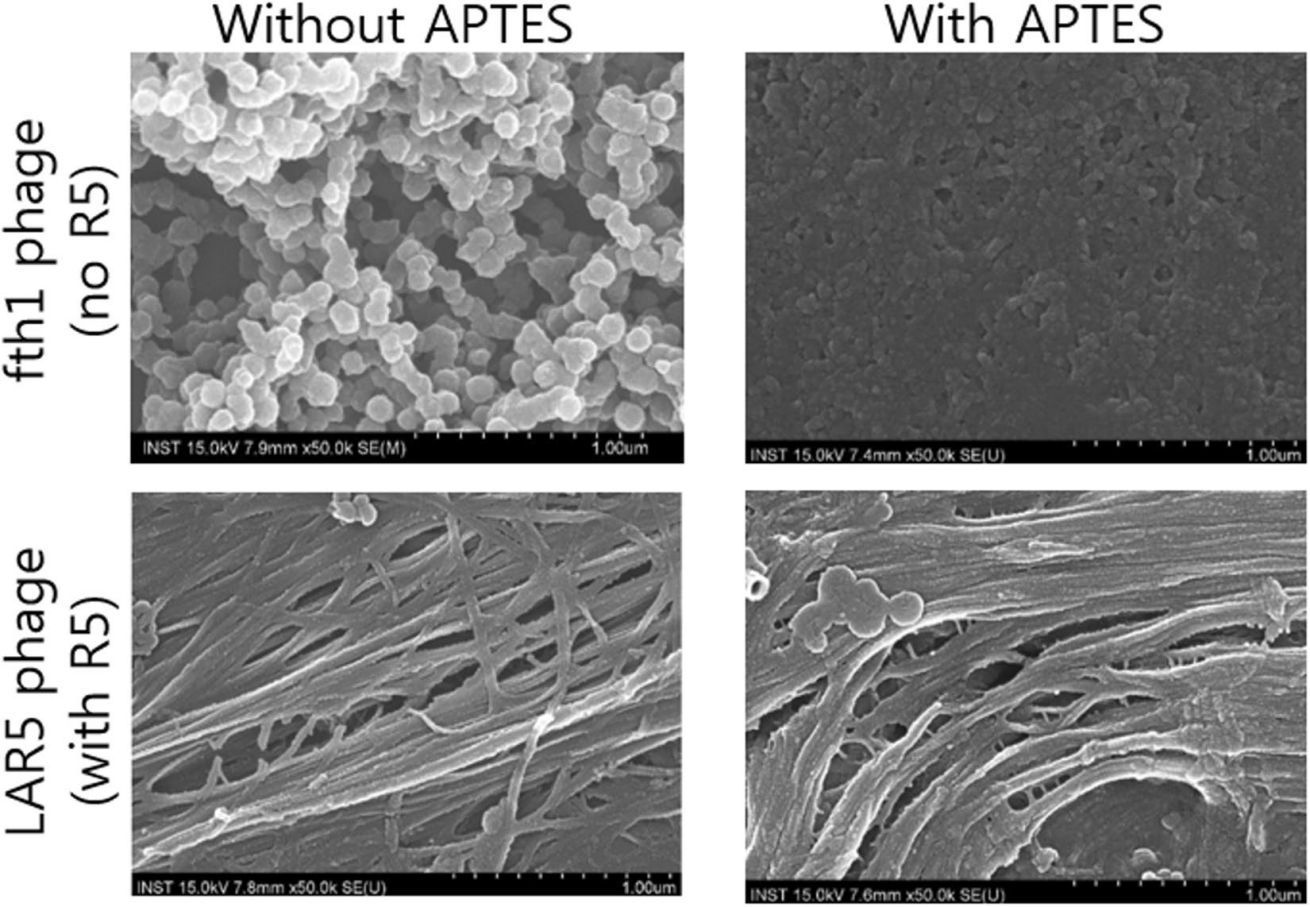
Comparison of phage type with presence or absence of APTES. SEM images of fth1 phage (top) and R5 bearing phage (bottom) in the presence of APTES (right) and without APTES (left) to observe the effects upon silicification.

To observe the role of the R5 phage in replacing the APTES and by serving as a natural structure for templating of TMOS for silica nanofiber formation, we examined the LAR5 phage possessing the R5 silaffin-construct on the p8 major coat as well as the fth1 phage having only wildtype p8. From Fig. 4 (using 900 uL of 1 mg/mL of phage and 100 uL of 50 mM TMOS), we can observe that R5 significantly helps the formation of fiber-like silica on LAR5 and moreover that the LAR5 phage are capable of producing silica nanofiber morphologies regardless of the presence of APTES confirming that LAR5 phage does not require APTES to form silica nanofibers. In contrast, the fth1 phage showed no clear silica nanofiber formation in 15 minutes reaction with or without APTES but rather exhibited small spherical morphologies; however, we find that fth1 silicification is enhanced when APTES is present.

## Discussion

In looking at the impact of implementing the R5 silaffin peptide sequence on the p8 coat of the filamentous bacteriophage, we reveal a means for generating silica nanofiber morphologies without the need for addition of APTES. Nonetheless, the addition of APTES does enhance the silicification process of phage without the R5 silaffin peptide and can change the morphology to some extent for the LAR5 phage with possessed the R5 construct on the p8 coat. The fth1 phage revealed a more spherical shape which could suggest the initial growth of silica particles from isolated distant locations, as we observed a significant increase in silica formation with time. In contrast, the LAR5 phage exhibited very closely linked beads on a string morphology to afford a linear nanofiber. This may lead to our assessment of how LAR5 phage are capable of generating silicified nanofibers without the need for addition of APTES in that the R5 phage can serve as a uniform template throughout the phage particle for simultaneous growth of particle from multiple locations throughout the phage. These results are meaningful in comparison to the fth1 phage which requires APTES since it does not possess the R5 moiety. In addition, the LAR5 phage also showed unique patterns unlike prior phage-based silicification approaches. Additional recent works have also shown the use of APTES-free mineralization using the R5 peptide. Specifically, the use of genetic engineering to create an R5 peptide fusion on silk to generate silk-silica biomaterials which stimulate osteogenesis^24^, an R5 fusion with protein A for generation of a solid matrix of silica displaying protein A units with applications to affinity resins or immunoprecipitation^25^, and a functional mimic of the silaffin tandem repeats made by peptide synthesis to generate assembled protein fibers for silica wire patterning without anchoring to a natural protein^26^. Our ability to make linear fiber-shaped nanoscale hybrid structures in an APTES-free system also confirmed that the R5 peptide present on the LAR5 can be used as a genetically integrated supporting material for silica nanofiber synthesis using the phage as a fibrous scaffold.

In summary, we presented a method for silicification on a bacteriophage that successfully yielded filamentous nanoscale hybrid structures. Importantly, unlike previously reported fd phage methods, this procedure can be conducted without 3-aminopropyl triethoxysilane owing to the presence of the R5 peptide on the major coat of the phage for templating of TMOS. In addition, we used a lower concentration of phage than existing approaches previously reported^27^, and we expect that such hybrid structures could be generated at even lower concentrations. We anticipate that the silica nanostructure fabrication process presented here would facilitate sustainable development because of the environmentally benign processing techniques. As with existing nanofiber silica applications, there may exist potential use of these structures for energy storage or catalytic templates.

## Methods

### Plasmid construction and transformation

A ‘type 88’ vector known as fth1, which contains two p8 genes within one bacteriophage’s genome, was used^28^. The fth1 vector was kindly provided by the laboratory of Prof. Jonathan Gershoni. The fth1 vector was transformed into chemically competent *Escherichia coli* TG1 for amplification. E. *coli* TG1/fth1 was grown at 37 °C and 150 rpm in LB medium (10 g/L tryptone, 5 g/L yeast extract, and 5 g/L NaCl) containing 25 μg/ml tetracycline antibiotic. Fth1 plasmid DNA was extracted after amplification in *E. coli* TG1 by using an AccuPrep^®^ Nano-Plus Plasmid Mini Extraction Kit (Bioneer, Korea). The fth1 vector contained the wild-type p8 gene and a location for a second p8 gene blocked by a trpA transcription terminator between two *Sfi*I restriction enzyme sites. An STS peptide, which existed before the transcriptional terminator of the inducible p8 gene, was removed for construction of the LAR5 (linker attached silaffin R5) vector. The extracted plasmid DNA from *E. coli* TG1/fth1 was digested with *Sfi*I restriction enzyme and resolved by gel electrophoresis as a band of size 8184 bp, which was collected with an AccuPrep^®^ Gel Purification Kit (Bioneer, Korea). The linker-attached silaffin R5 peptide gene was synthesized by a DNA synthesis service (Bioneer, Korea). The synthetic DNA for the gene was flanked by two *SfiI* restriction enzyme sites, along with an additional four sequences that brought the sequence in frame. The vector, which contained the synthetic R5 gene, was digested with *Sfi*I restriction enzyme and purified by gel electrophoresis, and the band containing sticky ends (approximately 100 bp in size) was collected with an AccuPrep^®^ Gel Purification Kit (Bioneer, Korea). After digestion and purification, the two DNA fragments were ligated using Mighty Mix (Takara, Japan). Specifically, the DNA insert for the linker-attached silaffin R5 peptide, having the amino acid sequence Ser-Ser-Lys-Lys-Ser-Gly-Ser-Tyr-Ser-Gly-Ser-Lys-Gly-Ser-Lys-Arg-Arg-Ile-Leu-Gly-Gly-Ser-Gly-Ser-Ser, was ligated between the two *Sfi*I cleavage sites to generate the final vector, referred to as LAR5, which was transformed into *E. coli* DH5α competent cells (Solgent, Korea) according to the manufacturer’s protocol. The transformant was maintained in this stable cell line for record keeping purposes, and the LAR5 vector was also transformed into *E. coli* TG1 using electroporation. The LAR5 vector was then examined for expression of the linker-attached silaffin R5 peptide on the phage major coat protein, and the silicification capabilities were assessed. The primers used in the construction and confirmation of the LAR5 phage vector are provided in Table 1.

**Table 1:** Primer sequences used for construction and verification of LAR5 phage from fth1.

Primer	Sequence
LAR5-F	5′-TAA AGG CCA ACG TGG CCA ATC TTC TAA GAA GTC TGG TTC TTA CTC TGG-3′
LAR5-R	5′-ACT AGG CCC CAG AGG CCT CGC TAG AAC CGC TGC CAC CCA AAA TTC TTC-3′
LAR5_con-F1	5′-ATA TCT GAA GGT TGG TTA GAT TTC CCT GTT-3′
LAR5_con-R1	5′-AAT CTC CAA AAA AAA AGG CTC CAA AAG GAG-3′
LAR5_con-F2	5′-CTA GCC ATC AGA TCT GCA CTG CT-3′
LAR5_con-R2	5′-CGT AGC CTA TGT ACT CAG TTG CG-3′

### LAR5 phage expression, purification, and quantification

The *E. coli* TG1/LAR5 was grown at 37 °C and 150 rpm in LB medium containing 25 μg/ml tetracycline. When the culture reached an optical density of 0.4 at 600 nm, 5 mM isopropyl β-D-1-thiogalactopyranoside (IPTG, Gold-bio, USA) was added, and cultures were incubated overnight at 25 °C and 150 rpm for effective production of the LAR5 phage. After overnight culture, the sample was centrifuged at 4000 rpm and 4 °C. Approximately 80% of the supernatant was harvested and mixed with a pre-made solution of 2.5 M NaCl/20% w/v polyethylene glycol (PEG-8000, Sigma-Aldrich, USA) solution. The mixture was then incubated at 4 °C and centrifuged as described above to obtain the precipitated phage. The recovered pellet was resuspended with phosphate buffer and vortexed for 10 minutes. After the same process was repeated three times, the mixture was centrifuged at 4 °C and 13000 rpm for 10 minutes to obtain the pure LAR5 phage. In addition, an ultra-centrifugation process was used to obtain a highly purified sample. The pure LAR5 phage solution was mixed with 0.75 g/ml cesium chloride. The mixture was centrifuged in an ultracentrifuge (Sorvall WX 80+ Ultracentrifuge, Thermo fisher, USA) using a fixed angle T-1250 rotor at 45000 rpm at 4 °C for approximately 24 hours. After spinning, a white-gray layer was observed in the tube corresponding to the phage. A syringe needle was used to collect the phage layer, and the collected phage sample was dialyzed against sterile phosphate buffer before storage at 4 °C until further use^29^. The quantity of LAR5 phage was measured with an UV/Vis absorbance spectrophotometer microplate reader (SPECTROstar Nano, BMG Labtech, Germany). The reference was phosphate buffer, and the sample was measured at 269 nm. The number of LAR5 phages was calculated on the basis of these measured values^22^.

### Mass analysis by liquid chromatography/mass spectroscopy

The existence of the linker-attached silaffin R5 peptide was verified by liquid chromatography-tandem mass spectrometry (Q-TOF 5600 LC-MS/MS system, AB SCIEX, USA) through electrospray ionization (ESI) in positive mode. The samples were analyzed with a 5600 Q-TOF LC/MS/MS system (AB Sciex, Foster City, CA) using an Ultimate 3000 RSLC HPLC system (Dionex), including a degasser, autosampler, Diode array detector and binary pump. The LC separation was performed on an Agilent C8 column (Agilent Zorbax 300SB-C8, 2.1*50 mm, 3.5 µm, 300 A) with mobile phase A (0.1% formic acid in water) and mobile phase B (0.1% formic acid in acetonitrile). The flow rate was 0.25 ml/min. The linear gradient was as follows: 0–4 min, 99% A; 4.1–8.0 min, 50% A; and 8.1–13.0 min, 5% A. The autosampler was set at 5 °C. The injection volume was 20 μl. Mass spectra were acquired under positive electrospray ionization (ESI) with an ion spray voltage of 4500 V. The source temperature was 450 °C. The curtain gas, ion source gas 1, and ion source gas 2 were 35, 65, and 55 psi, respectively.

### Biosilicification

The silicification was implemented using 900 μl of LAR5 phage (concentration as indicated in the test above of either 2 mg/mL for the initial tests or 0.1 mg/mL for further tests) and 100 μl of 50 mM tetramethyl orthosilicate in distilled water (TMOS, Sigma-aldrich, USA), which was pre-hydrolyzed in 1 mM HCl. After the two components were mixed, the samples were vortexed for 1 min, incubated for 1 min on ice and then left at room temperature for 13 min. The silicification was executed under mild conditions, and the products were harvested by centrifugation at 4 °C and 13000 rpm^8^.

### Scanning electron microscopy imaging of the silicified phage

The surface morphology was analyzed by field emission scanning electron microscopy (Nova NanoSEM 450 scanning electron microscope, FEI Company, USA) at 15 kV after silicification with purified LAR5 phage and tetramethyl orthosilicate (TMOS, Sigma-Aldrich, USA). A specimen was prepared on a piece of cleaned glass and then fully dried. The entire dried sample was coated with platinum by ion sputtering (E-1045 ion sputter, Hitach, Japan) and secured using adhesive silver paste.

### Data availability

All data generated or analysed during this study are included in this published article.

## References

[1] Hench LL (1991). Bioceramics: From Concept to Clinic. Journal of the American Ceramic Society.

[2] Patwardhan SV (2012). Chemistry of aqueous silica nanoparticle surfaces and the mechanism of selective peptide adsorption. J Am Chem Soc.

[3] Ibrahim IA, Zikry AAF, Sharaf MA (2010). Preparation of spherical silica nanoparticles: Stober silica. Journal of American Science.

[4] He T, Abbineni G, Cao BR, Mao CB (2010). Nanofibrous Bio-inorganic Hybrid Structures Formed Through Self-Assembly and Oriented Mineralization of Genetically Engineered Phage Nanofibers. Small.

[5] Nam KT (2006). Virus-Enabled Synthesis and Assembly of Nanowires for Lithium Ion Battery Electrodes. Science.

[6] Liu N, Huo K, McDowell MT, Zhao J, Cui Y (2013). Rice husks as a sustainable source of nanostructured silicon for high performance Li-ion battery anodes. Sci Rep.

[7] Ryu J, Kim SW, Kang K, Park CB (2010). Mineralization of self-assembled peptide nanofibers for rechargeable lithium ion batteries. Adv Mater.

[8] Nam DH, Won K, Kim YH, Sang BI (2009). A novel route for immobilization of proteins to silica particles incorporating silaffin domains. Biotechnol Prog.

[9] Kroger N, Deutzmann R, Sumper M (1999). Polycationic peptides from diatom biosilica that direct silica nanosphere formation. Science.

[10] Otzen D (2012). The role of proteins in biosilicification. Scientifica (Cairo).

[11] Sumper M, Kroger N (2004). Silica formation in diatoms: the function of long-chain polyamines and silaffins. Journal of Materials Chemistry.

[12] Kroger N, Deutzmann R, Bergsdorf C, Sumper M (2000). Species-specific polyamines from diatoms control silica morphology. Proc Natl Acad Sci USA.

[13] Lechner CC, Becker CF (2014). A sequence-function analysis of the silica precipitating silaffin R5 peptide. J Pept Sci.

[14] Voelcker NH, Tajuddin I, Voelcker N, Mitchell J (2006). Silica nanostructure formation from synthetic R5 peptide. SPIE International Symposium on Smart Materials, Nano-and Micro-Smart Systems.

[15] Senior L (2015). Structure and function of the silicifying peptide R5. J. Mater. Chem. B.

[16] Altintoprak K (2015). Peptide-equipped tobacco mosaic virus templates for selective and controllable biomineral deposition. Beilstein journal of nanotechnology.

[17] Patwardhan SV, Mukherjee N, Clarson SJ (2001). The use of poly-L-lysine to form novel silica morphologies and the role of polypeptides in biosilicification. Journal of Inorganic and Organometallic Polymers.

[18] Li D, Mathew B, Mao C (2012). Biotemplated Synthesis of Hollow Double-Layered Core/Shell Titania/Silica Nanotubes under Ambient Conditions. Small.

[19] Wang F, Li D, Mao C (2008). Genetically modifiable flagella as templates for silica fibers: from hybrid nanotubes to 1D periodic nanohole arrays. Advanced functional materials.

[20] Iannolo G, Minenkova O, Petruzzelli R, Cesareni G (1995). Modifying Filamentous Phage Capsid: Limits in the Size of the Major Capsid Protein. j. Mol. Biol..

[21] Sidhu SS, Weiss GA, Wells JA (2000). High Copy Display of Large Proteins on Phage for Functional Selections. j. Mol. Biol..

[22] Chung WJ (2011). Biomimetic self-templating supramolecular structures. Nature.

[23] Choi O (2011). A biosensor based on the self-entrapment of glucose oxidase within biomimetic silica nanoparticles induced by a fusion enzyme. Enzyme Microb Technol.

[24] Mao C, Wang F, Cao B (2012). Controlling nanostructures of mesoporous silica fibers by supramolecular assembly of genetically modifiable bacteriophages. Angew Chem Int Ed Engl.

[25] Petrenko GPSaVA (1997). Phage Display. Chemical Society reviews.

[26] Martín-Moldes, Z. *et al*. Intracellular Pathways Involved in Bone Regeneration Triggered by Recombinant Silk–Silica Chimeras. *Advanced Functional Materials* **2017**, 1702570. 10.1002/adfm.201702570 PMC6101667 30140193

[27] Park KS, Ki MR, Yeo KB, Choi JH, Pack SP (2017). Design of Bio-inspired Silica-encapsulated Protein A for Improved Immunoprecipitation Assays. Biochemical Engineering Journal.

[28] Bella A, Ray S, Ryadnov M (2017). Linear and orthogonal peptide templating of silicified protein fibres. Organic & Biomolecular Chemistry.

[29] Unwin RR (2015). DNA driven self-assembly of micron-sized rods using DNA-grafted bacteriophage fd virions. Physical Chemistry Chemical Physics.

